# The Respiratory Arsenite Oxidase: Structure and the Role of Residues Surrounding the Rieske Cluster

**DOI:** 10.1371/journal.pone.0072535

**Published:** 2013-08-30

**Authors:** Thomas P. Warelow, Muse Oke, Barbara Schoepp-Cothenet, Jan U. Dahl, Nicole Bruselat, Ganesh N. Sivalingam, Silke Leimkühler, Konstantinos Thalassinos, Ulrike Kappler, James H. Naismith, Joanne M. Santini

**Affiliations:** 1 Institute of Structural and Molecular Biology, University College London, London, United Kingdom; 2 Centre for Biomolecular Sciences, University of St Andrews, St Andrews, United Kingdom; 3 Laboratoire de Bioénergétique et Ingénierie des Protéines, BIP/CNRS, UMR7281, AMU, Marseille, France; 4 Universität Potsdam, Institut für Biochemie and Biologie, Potsdam, Germany; 5 School of Chemistry and Molecular Biosciences, The University of Queensland, St. Lucia, Queensland, Australia; Instituto de Tecnologica Química e Biológica, UNL, Portugal

## Abstract

The arsenite oxidase (Aio) from the facultative autotrophic *Alphaproteobacterium Rhizobium* sp. NT-26 is a bioenergetic enzyme involved in the oxidation of arsenite to arsenate. The enzyme from the distantly related heterotroph, *Alcaligenes faecalis*, which is thought to oxidise arsenite for detoxification, consists of a large α subunit (AioA) with *bis*-molybdopterin guanine dinucleotide at its active site and a 3Fe-4S cluster, and a small β subunit (AioB) which contains a Rieske 2Fe-2S cluster. The successful heterologous expression of the NT-26 Aio in *Escherichia coli* has resulted in the solution of its crystal structure. The NT-26 Aio, a heterotetramer, shares high overall similarity to the heterodimeric arsenite oxidase from *A. faecalis* but there are striking differences in the structure surrounding the Rieske 2Fe-2S cluster which we demonstrate explains the difference in the observed redox potentials (+225 mV *vs.* +130/160 mV, respectively). A combination of site-directed mutagenesis and electron paramagnetic resonance was used to explore the differences observed in the structure and redox properties of the Rieske cluster. In the NT-26 AioB the substitution of a serine (S126 in NT-26) for a threonine as in the *A. faecalis* AioB explains a −20 mV decrease in redox potential. The disulphide bridge in the *A. faecalis* AioB which is conserved in other betaproteobacterial AioB subunits and the Rieske subunit of the cytochrome *bc*_1_ complex is absent in the NT-26 AioB subunit. The introduction of a disulphide bridge had no effect on Aio activity or protein stability but resulted in a decrease in the redox potential of the cluster. These results are in conflict with previous data on the betaproteobacterial AioB subunit and the Rieske of the *bc*_1_ complex where removal of the disulphide bridge had no effect on the redox potential of the former but a decrease in cluster stability was observed in the latter.

## Introduction

Aerobic arsenite oxidation in the Alphaproteobacterium *Rhizobium* sp. NT-26 is an energy-generating process where the electron donor, arsenite, is oxidized to the less toxic arsenate and this is coupled to the reduction of oxygen to water [1]. NT-26 can oxidize arsenite either autotrophically with carbon dioxide as the sole carbon source or heterotrophically, where yeast extract is used as the source of carbon [1]. Aerobic arsenite oxidation is catalysed by arsenite oxidase (Aio) [2] which is thought to be an ancient bioenergetic enzyme that was present in the last universal common ancestor prior to the divergence of the Bacteria and Archaea [3], [4]. Aio consists of two heterologous subunits, a large (93 kDa) catalytic subunit (AioA) which contains the molybdenum cofactor (Moco) at the active site and a 3Fe-4S cluster, and a small subunit (14 kDa) subunit (AioB) which contains a Rieske 2Fe-2S cluster [5]. The Aio belongs to the dimethylsulphoxide (DMSO) reductase enzyme family of molybdoenzymes but is unusual in that it’s the only member of this family to contain a 3Fe-4S cluster and a Rieske subunit [6].

The X-ray crystal structure of the Aio from the distantly related (i.e. a member of the *betaproteobacteria*) heterotrophic arsenite oxidiser *Alcaligenes faecalis* has been determined as a heterodimer (α_1_β_1_) [6]. Molybdenum (Mo) is located in a highly solvated funnel-like cavity in the AioA subunit and is coordinated by two antiparallel molybdopterin guanine dinucleotide cofactors (*bis*-MGD), three water molecules and one oxo ligand. Several protein residues coordinate the *bis*-MGD in an extensive network of hydrogen bonds and salt bridges. The Mo atom is not coordinated to the protein unlike what has been seen in other members of the DMSO reductase family whose crystal structures have been determined, and which have a Ser, Cys, Asp or SeCys, contributing a ligand to the Mo atom [7]. Three water molecules bind to H195, E203, R419 and H423, and make direct contact with the Mo = O group. A manual fit of the arsenite substrate suggests that the three water molecules occupy the substrate-binding site. The 3Fe-4S cluster in AioA (approximately 15 Å from the Mo atom) is coordinated by the conserved motif (Cys21-X_2_-Cys24-X_3_-Cys28-X_70_-Ser99 in AioA). Similar to other Rieske- and Rieske-type proteins, the AioB subunit of *A. faecalis* contains a sequence motif (Cys60-X-His62-X_15_-Cys78-X_2_-His81) which binds the Rieske 2Fe-2S cluster. The arsenite is oxidized to arsenate at the Mo-site in AioA, reducing the Mo from +VI to +IV. Since the 3Fe-4S cluster is a one electron acceptor it is assumed that it accepts one electron from the Mo-pterin and then transfers an electron at a time to the Rieske 2Fe-2S cluster of the AioB subunit. The electron is then transferred from the Rieske centre to a physiological electron acceptor (e.g. cytochrome *c*) and finally, in aerobes, to a cytochrome oxidase where oxygen is reduced to water [8]–[11].

NT-26 Aio was purified as a heterotetramer (α_2_β_2_) with a native molecular mass of 219 kDa [5]. The Aio subunits from *A. faecalis* and NT-26 share 48% identity. Both the 3Fe-4S-binding motif and the predicted arsenite-binding residues are conserved in the NT-26 AioA and may play similar roles. The AioB subunits are 49% identical and the 2Fe-2S-binding motif is also conserved. One of the most striking differences is that the *A. faecalis* AioB possesses a disulphide bridge - C65–C80 - which connects the two loops at the Rieske centre whereas the equivalent residues in the NT-26 AioB are F108 and G123.

Both the *A. faecalis* and NT-26 arsenite oxidases share common redox and spectral properties when studied by EPR. In both enzymes, no Mo (V) signal has been detected suggesting that the only stable redox states of this centre are Mo(IV) and Mo(VI), and the redox potential of the 3Fe-4S cluster has been determined to be +270 mV for both enzymes [5], [12]. A significant difference however has been observed between the redox potentials of the AioB Rieske 2Fe-2S clusters, with +130/160 mV in *A. faecalis* and +225 mV in NT-26 [12], [13]. This compares to +300 mV for the redox potential of the Rieske cluster of the *Rhodobacter sphaeroides bc*_1_ complex [12]. Several groups have suggested that the high redox potentials of the *bc*_1_ complex Rieske clusters are due to the cumulative effects of a disulphide bridge and hydrogen bonds from Tyr and Ser residues [14]–[18].

In this study, we report the first heterologous expression and X-ray crystal structure of the arsenite oxidase from the autotrophic arsenite-oxidising bacterium NT-26. We have compared the structure with that from *A. faecalis* and other *bis*-MGD-containing enzymes. We have also used a combination of site-directed mutagenesis and EPR to understand the role of residues surrounding the Rieske cluster and the role of the disulphide bridge on the redox potential of the cluster.

## Materials and Methods

### Bacterial Strains, Plasmid and Growth Conditions

*E. coli* strains DH5α [19], JM109λ*pir* [20], RK4353 [21] and C43 [22] were used for expression of the NT-26 Aio. The vector pPROEX-HTb (Invitrogen) was used for expression. All expression conditions involved growing *E. coli* in Luria Bertani (LB) broth containing 100 µg/ml ampicillin either aerobically (170 rpm with 1∶5 ratio liquid to head space) or anaerobically with nitrate (14 mM) or DMSO (14 mM) as electron acceptors and sodium lactate (20 mM) as the electron donor.

### Cloning and Expression

The NT-26 *aioB* and *aioA* (*aioBA*) genes were amplified without the native twin-arginine translocation (Tat) leader sequence using the following primers:

Forward 5′-GCGAATTCAAGCTACCGCGGCGGCAGGGGTC-3′ and Reverse 5′-GCCTGCAGTCAAGCCGACTGGTATTCTTTCGA-3′. The restriction enzymes *Eco*RI and *Pst*I (underlined above) were used for cloning into the expression vector, pPROEX-HTb. The *aioBA* clone sequence was confirmed. The pPROEX-HTb carrying the *aioBA* genes was transformed into a variety of *E. coli* strains to determine which one gave optimal expression. A variety of IPTG (isopropyl β-D-1-thiogalactopyranoside) and sodium molybdate concentrations as well as induction times were also tested. The final optimum expression conditions used for purification of the Aio involved growing DH5α aerobically at 21°C for 24 h in LB containing 40 µM IPTG and 1 mM sodium molybdate.

### Site-directed Mutagenesis

The primers used to create point mutations in the *aioB* gene are shown in Table S1. Mutants were generated using the Agilent Quick Change II XL site-directed mutagenesis kit according to the manufacturer’s instructions. Mutations were confirmed by sequencing. The double mutant was created by sequential single mutations. Mutants were expressed and purified as described below for the wild type enzyme.

### Purification of the Recombinant Arsenite Oxidases

The recombinant arsenite oxidases were purified from DH5α using a combination of affinity and size exclusion chromatography. Cells were harvested by centrifugation at 9,700 *g* for 10 min. The cell pellets were pooled and washed by suspending in binding buffer (20 mM potassium phosphate, 500 mM sodium chloride, 20 mM imidazole, pH 7.3) at 10 ml/g wet weight cells and centrifuged at 12,000 *g* for 15 minutes. The cell pellet was resuspended in binding buffer (10 ml/g wet weight cells). The *E. coli* cells were disrupted by a single passage through a French pressure cell (12,000 psi) and the cell debris removed by centrifugation at 30,000 *g* for 30 minutes. The supernatant was loaded onto a 1 ml GraviTrap pre-packed Ni charged affinity chromatography column (GE Healthcare) as per the manufacturer’s instructions except with one minor modification; the wash volume used was 120 ml. The eluent was desalted in 50 mM MES (pH 5.5) buffer resulting in the precipitation of protein(s) which were removed by centrifugation at 10,000 *g* for 5 min. The supernatant was filtered through a 0.22 µm filter (Millipore), concentrated using a Vivaspin 20 (MWCO 100,000) (GE Healthcare) centrifugal concentrator and loaded onto a Superdex 200 10/300 gel filtration column (GE Healthcare) pre-equilibrated with 50 mM MES, 100 mM NaCl, pH 5.5 buffer. Chromatography was carried out at a flow rate of 0.3 ml/min. The 0.25 ml fractions containing Aio activity were pooled and concentrated using a Vivaspin 20 centrifugal concentrator (MWCO 100,000). For crystallization the His-tag encoded by the vector was removed using rTEV.

Confirmation of the native molecular mass of the recombinant Aio was done using a Superdex 200 10/300 gel filtration (GE Healthcare) chromatography column with a calibration curve created using a gel filtration HMW calibration kit (GE Healthcare). Chromatography conditions used were as described by the manufacturer with a flow rate of 0.3 ml/min.

### Enzyme Assays

Arsenite oxidase enzyme assays were done as described previously [1] at 25°C using the artificial electron acceptor, 2,6-dichlorophenolindophenol (DCPIP) in 50 mM MES (pH 5.5; optimum buffer). The results of the kinetics of the wild type enzyme are from at least two independent experiments with at least two replicates for each arsenite concentration tested. The specific activities calculated for the mutants with 2.5 mM arsenite are an average of two independent experiments with at least two replicates per experiment. An activity temperature profile was conducted at a range of temperatures controlled with a Varian Cary dual cell Peltier accessory as described previously [23]. Each data point represents a minimum of three replicates. Protein concentrations were determined using spectroscopic absorbance readings at 280 nm using a NanoDrop 2000 spectrophotometer (Thermo) and a predicted molar absorbance coefficient (ExPASy, Swiss Institute of Bioinformatics) based on the calculated protein extinction coefficient (280 nm) using the Edelhoch method [24], with the extinction coefficients for Trp and Tyr determined by Pace *et al.* [25].

### Cofactor Analysis

Metal analysis was performed using a PerkinElmer Life Sciences Optima 2100DV inductively coupled plasma optical emission (ICP-OES) spectrometer (Fremont, CA, USA). 500 µL of protein samples (5 µM) were incubated overnight in a 1∶1 mixture with 65% nitric acid (Suprapur, Merck, Darmstadt, Germany) at 100°C and diluted in a total volume of 5 ml with water. As reference, the multi-element standard solution XVI (Merck) was used. For nucleotide analysis, GMP was released from *bis*-MGD by incubation in 5% (v/v) sulphuric acid for 15 min. GMP released during the incubation was separated by HPLC using a C18 reverse-phase column (4.6×250 mm, ODS Hypersil column, particle size of 5 µM; Thermo Scientific) equilibrated in 50 mM diammonium phosphate (pH 2.5), 1% methanol at an isocratic flow rate of 1 ml/min. AMP, GMP and CMP concentrations were quantified by using AMP, GMP and CMP standard solutions.

### Mass Spectrometry

For confirmation of the presence of the disulphide bridge the β F108C/G123C mutant was buffer exchanged into 250 mM ammonium acetate at pH 7.5, concentrated to 10 µM using Amicon Ultra 0.5 ml centrifugal filters (Millipore UK Ltd, Watford UK) and then diluted 2∶1 in denaturing buffer (50∶50 water:methanol). Mass spectrometry experiments were carried out on a Synapt HDMS (Waters Ltd, Manchester, UK) QTOF mass spectrometer [26] and 2.5 µl aliquots of protein samples were delivered to the mass spectrometer by means of nanoESI using gold-coated capillaries, prepared in house. Typical instrumental parameters were as follows: source pressure 6 mbar, capillary voltage 1.20 kV, cone voltage 40 V, trap energy 10 V, transfer energy 8 V, and trap pressure 3.6×10–2 mbar. Data acquisition and processing were carried out using MassLynx (ver. 4.1) software (Waters Corp., Milford, MA, USA). Mass deconvolution was carried out using the Maximum Entropy algorithm available as part of the MassLynx software.

### Structural Biology

Crystals were obtained from sitting drop vapour diffusion against a reservoir of 0.1 M Hepes sodium pH 7.5, 2% polyethylene glycol (PEG) 400, 2.0 M ammonium sulphate. Data were recorded at the ESRF ID23-1 to a resolution of 2.7 Å from a single crystal bathed in crystallization mother liquor with 10% glycerol, cooled to 100 K prior to data collection (Table S2). Data were processed using MOSFLM/SCALA [27], [28]. The structure was solved by molecular replacement [with the starting model of the *A. faecalis* high resolution structure (PDB 1 g8k – see RCSB www.pdb.org)] with separate α chain and β chains (cut to polyalanine where residues were not conserved) using the program PHASER [29], [30] as implemented in CCP4 [31]. The structure was rebuilt to the correct sequence with a combination of the program BUCCANEER [32] and manual intervention with COOT [33]. The structure was refined with REFMAC5 [34] with TLS parameters [35], [36].

### EPR

EPR spectroscopy was performed on wild type and mutant enzymes obtained after the desalting step. Redox titrations were performed with approximately 2 mg of enzymes, at 15°C, pH 8, as described by Dutton [37] and adapted as described by Duval [12] in the presence of the following redox mediators at 100 µM: 1,4 *p*-benzoquinone, DCPIP, 2,5-dimethyl-*p*-benzoquinone, 2-hydroxy 1,2-naphthoquinone, 1,4-naphthoquinone. Reductive titrations were carried out using sodium dithionite, and oxidative titrations were carried out using ferricyanide. EPR spectra were recorded on a Bruker ElexSys X-band spectrometer fitted with an Oxford Instruments liquid-Helium cryostat and temperature control system.

The results of the wild type and mutant enzyme redox data were obtained with two separate enzyme preparations. Evaluation were performed on the *g* = 1.88 signal. A two-way ANOVA without replication showed significant variation among the four enzymes (*F*_ 3, 3_ = 26.51, *P* = 0.012).

## Results

### Characteristics of the Heterologously Expressed and Purified Arsenite Oxidase

**Figure 1:**
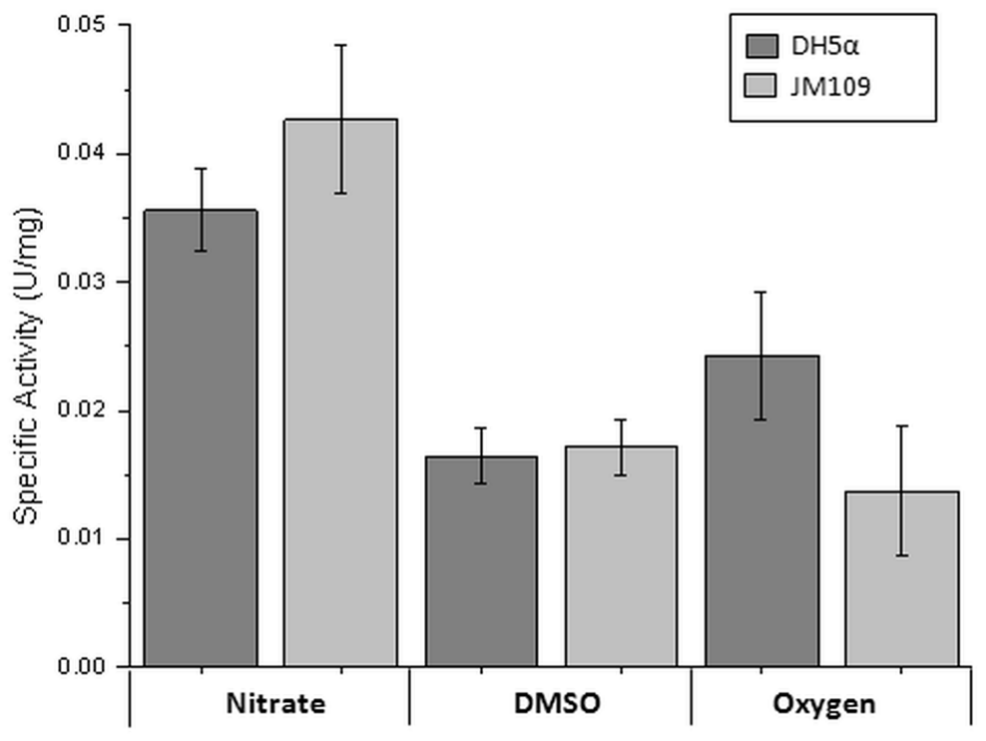
Comparison of arsenite oxidase activities in total cell extracts of *E. coli* strains. DH5α and JM109λ*pir* grown were grown with oxygen, nitrate and DMSO as terminal electron acceptors. Error bars represent the average of six individual experiments.

The *aioB* and *aioA* (designated *aioBA*) genes were cloned without the *aioB* Tat leader sequence into pPROEX-HTb (Invitrogen) under the control of the IPTG inducible *trc* promoter which allows for expression in the *E. coli* cytoplasm. A combination of different strains and growth conditions were tested to optimize Aio expression (Figure 1). Although the highest specific activity was detected when the enzyme was expressed under anaerobic conditions with nitrate as the terminal electron acceptor, the greater cell yield obtained from aerobic growth meant that aerobic conditions were chosen for further expression studies with DH5α as the host.

**Figure 2:**
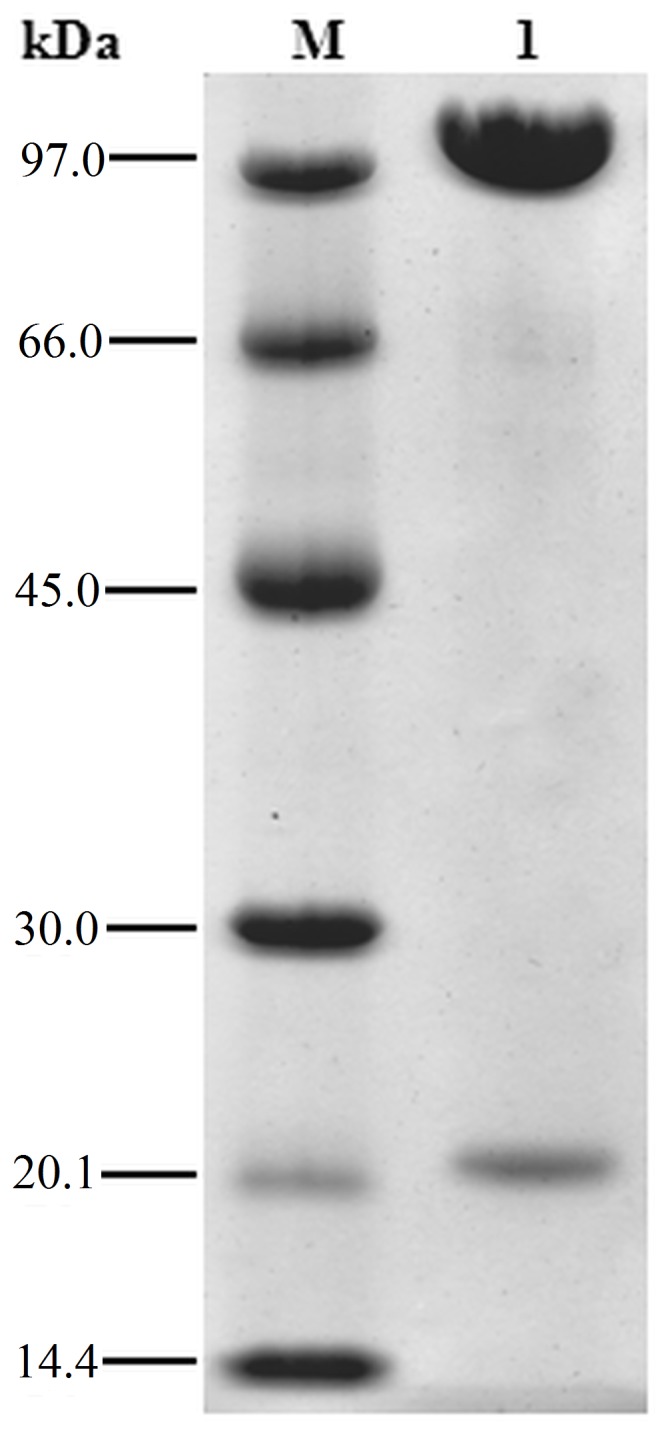
SDS-polyacrylamide gel (12%) of purified recombinant NT-26 Aio. M: Molecular weight marker: phosphorylase b (97 kDa), albumin (66 kDa), ovalbumin (45 kDa), carbonic anhydrase (30 kDa), trypsin inhibitor (20.1 kDa), α-lactalbumin (14.4 kDa) (GE Healthcare) 1: Purified recombinant AioBA, two subunits AioA (91.3 kDa) AioB with N-terminal His-tag (20 kDa).

Recombinant arsenite oxidase was purified from *E. coli* using a combination of Ni-NTA and size exclusion chromatography. The enzyme was enriched by about 20-fold (Table 1) with a yield of about 0.25 mg per g (wet weight) of cells. Based on SDS polyacrylamide gel electrophoresis, the enzyme was pure with both known Aio subunits present, namely AioA (91.3 kDa) and AioB with His tag (20 kDa) (Figure 2). Based on size exclusion chromatography the native molecular mass of the enzyme was 223 kDa which is consistent with the α_2_β_2_ oligomeric state of the native enzyme purified from NT-26 [5].

**Table 1:** Purification table of NT-26 recombinant arsenite oxidase.

Purification step	Total protein (mg)	Total activity (µmol^−1^ min^−1^)	Specific activity (µmol^−1^ min^−1^ mg^−1^)	Purification fold
Total cell extract	49.92	4.63	0.09	1.0
Ni-NTA	10.27	5.41	0.53	5.7
Buffer change/centrifugation	2.11	3.57	1.70	18.3
Superdex 200	1.19	2.18	1.85	19.9

Kinetics of the recombinant Aio was determined with DCPIP as the artificial electron acceptor as has been done previously for the native enzyme [5]. The enzyme was found to have similar kinetic properties to the native enzyme with a V*_max_* of 1.73±0.01 µmol^−1^ min^−1^ mg^−1^, K*_m_* of 68±4.8 µM and k*_cat_* of 3.6±0.25 s^−1^ compared with 2.4 µmol^−1^ min^−1^ mg^−1^, 61 µM and 4.3 s^−1^ (incorrectly calculated in the original paper) for the native enzyme [5].

The Mo and Fe contents of the recombinant Aio were quantified by ICP-OES and showed that the Aio was 83.1±1.3% saturated with Mo and 77.6±1.3% saturated with Fe (with respect to the 3Fe-4S in AioA and the 2Fe-2S cluster in AioB) (Table S3). The Moco in Aio was identified as the *bis*-MGD cofactor, since two GMP molecules were identified in relation to one Mo atom bound to the AioA catalytic unit. The GMP concentration was calculated to be 89.3±4.5% and no other nucleotides were found bound to AioBA (Table S2). In total, heterologously expressed AioBA in *E. coli* was saturated to at least 83% with the *bis-*MGD cofactor.

### Comparison of the NT-26 Arsenite Oxidase Structure to that of A. Faecalis and Other Molybdenum-containing Enzymes

**Figure 3:**
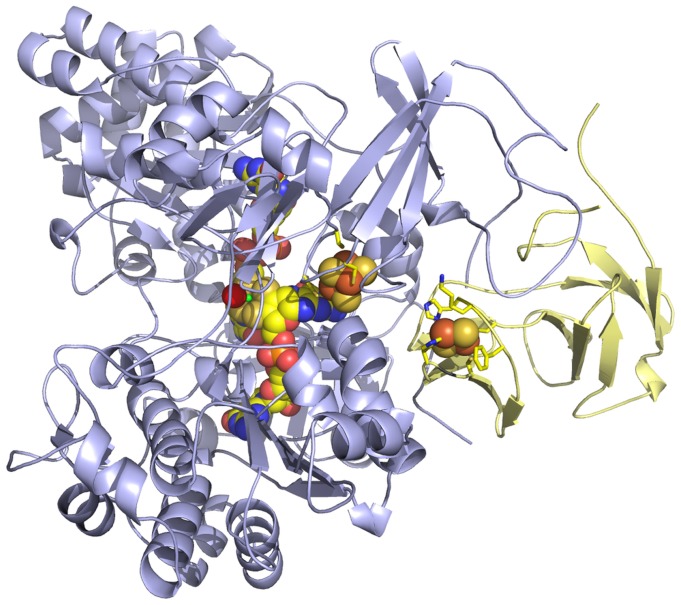
The heterodimeric structure of the Aio from NT-26. The Aio consists of an α (pale blue) and β chain (pale yellow). The pterin co-factor, 3Fe-4S, 2Fe-2S clusters are shown as space filling spheres. Residues which ligate the clusters are shown as sticks, as are the two residues surrounding the Rieske cluster (K106 and F108). Atoms are coloured iron orange, sulphur dark yellow, carbon bright yellow, molybdenum green, phosphorus bright orange, oxygen red, nitrogen blue.

The structure of the NT-26 Aio was solved to a resolution of 2.7 Å (Table S2). The asymmetric unit of arsenite oxidase contains four α and four β chains. The α chain contains a 3Fe-4S cluster and the *bis*-MGD cofactor, which contains a single Mo ion. This chain is ordered from A2 to S844. The β chain (Rieske subunit) contains a 2Fe-2S cluster and residues A44 to V175 are located in the experimental electron density. The enzyme is expressed without the Tat leader sequence from the β chain; however, an additional eight amino acids are present after rTEV cleavage (i.e. GAMGSGIQ). Each α chain has extensive interactions with a β chain forming a heterodimer (Figure 3). The fold of the α chain and of the β chain are both very similar to the corresponding chains in the enzyme from *A. faecalis* [6] and the relative arrangement of the domains with respect to each other is also conserved (Figure S1). Briefly, the α chain can be divided into four domains arranged in a pseudo tetrahedral arrangement with the Mo ion at the centre as is the case for all members of the DMSO family (Figure S2). The iron-sulphur cluster (in the case of Aio a 3Fe-4S cluster) is anchored to domain 1, a common feature of catalytic subunits of the DMSO reductase family, but contacts domain 3 (the helix T244 to R256). The Rieske domain has a six stranded antiparallel β barrel and a four stranded antiparallel β sheet which binds the 2Fe-2S cluster. The heterodimer of NT-26 (α and β subunits) superimposes with *A*. *faecalis* (1 g8j), 948 matching residues with an rmsd of 1.84 Å for cα atoms (the values for the individual chains are around 1 Å). A search of the PDB database reveals that the α subunit is closely related to the periplasmic nitrate reductase (NapA) from *Desulfovibrio desulfuricans* (PDB 2NAP, gives a superposition of the α subunit from NT-26 of 2.1 Å for 619 matching cα atoms), which has two pterin co-factors ligated to Mo and a 4Fe-4S cluster (not the 3Fe-4S seen in AioA). Other similar structures are all members of the DMSO reductase family. Some periplasmic nitrate reductases do possess a small β subunit but this is dissimilar to the typical Rieske fold of the AioB subunit [6], [38].

**Figure 4:**
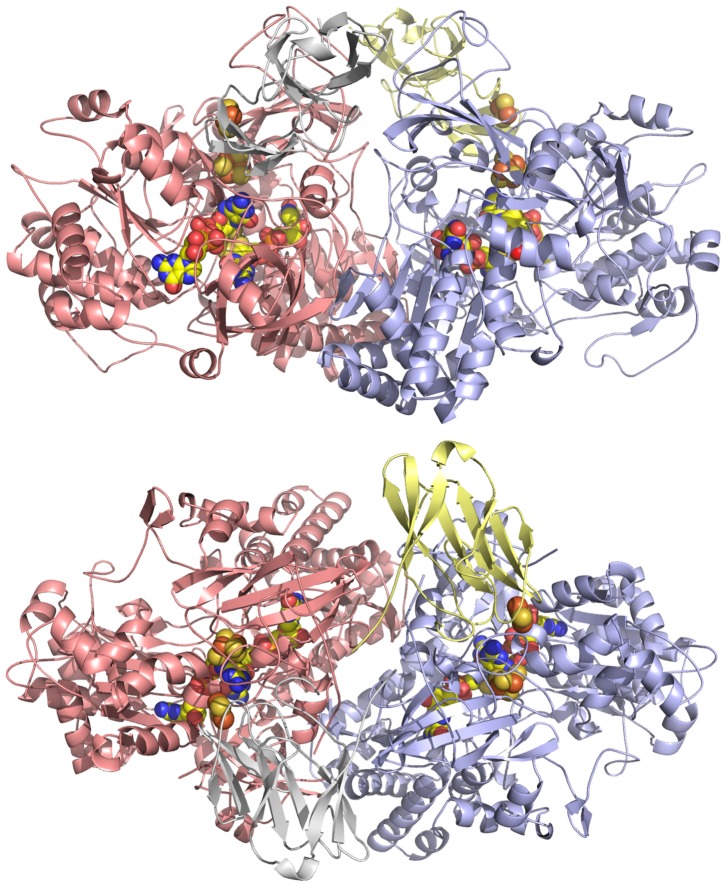
The heterotetrameric structure of the Aio from NT-26. The Aio consists of two αβ heterodimers. In the second heterodimer the α chain is coloured salmon, and the β chain grey.

Analysis of protein-protein interactions using PISA (Protein Interfaces, Surfaces and Assemblies) [39] shows that the α and β subunits that come together to form the heterodimer bury 8000 Å^2^ of exposed surface area. The same analysis reveals that the four heterodimers present in the asymmetric unit are arranged as two stable heterotetramers, and each heterotetramer buries 22000 Å^2^ of surface area (meaning the tetramerisation buries a further 6000 Å^2^ of surface area) (Figure 4). The contacts that stabilize the heterotetramer are between domains 2 of the α chain and to a lesser extent domain 1. There are also contacts between the N-termini of the β chains that contribute to the tetramer.

**Figure 5:**
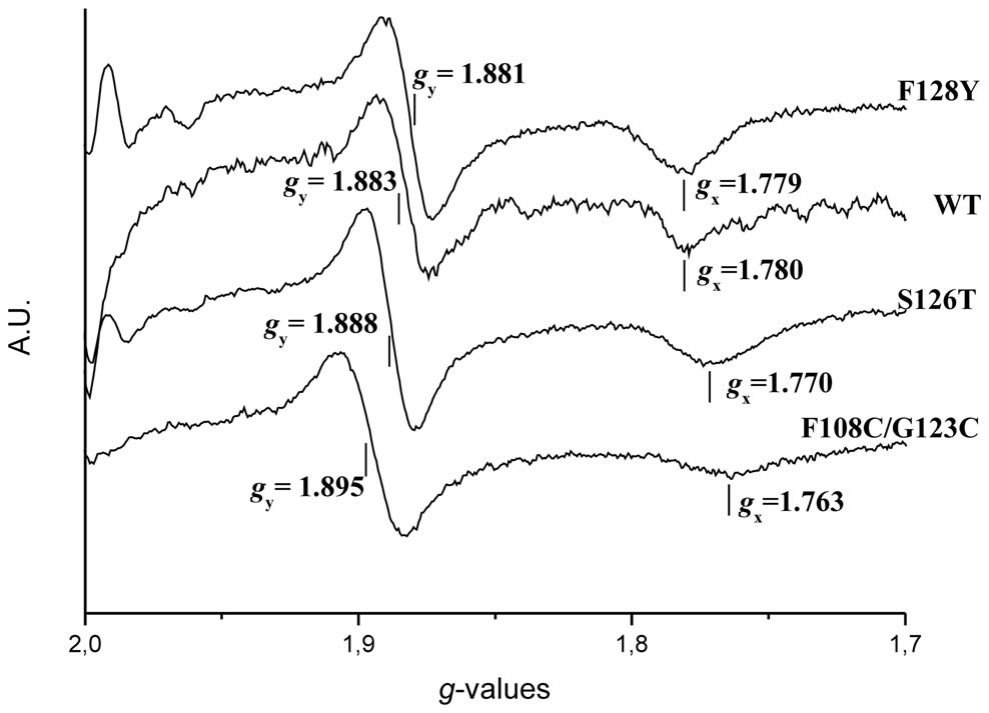
EPR properties of the WT and mutated Aio Rieske centres from NT-26. All spectra were recorded during titration on entirely reduced isolated complex under non-saturating conditions. Instrument settings: microwave frequency, 9,48 GHz; modulation amplitude 1.6 mT, temperature 15 K; microwave power, 6.3 mT.

Although the primary iron and sulphur ligands are the same in the Rieske cluster of *A. faecalis* and NT-26 AioB, there is a striking difference in the amino acids surrounding the cluster (Figure 5). In the homologous Rieske protein of the *bc*_1_ complex, the hydrogen bond network around the Rieske cluster has been shown to be responsible for the redox properties of the 2Fe-2S cluster (Figure 5). Residues β T61 and β M63 in *A. faecalis* AioB are replaced by β P104 and β K106 in the NT-26 AioB and these residues sit either side of the conserved H which ligates to the iron of the 2Fe-2S cluster. In the *bc*_1_ complex Rieske, these residues have been shown to be important for reactivity of the complex with quinone but not for the redox potential of the cluster [40]. These residues also don’t appear to be important for Rieske cluster redox potential in AioB, as the *Ralstonia* sp. S22 and NT-26 AioB subunits have similar redox potentials but the former like the *A. faecalis* AioB contains the T/M, instead of P/K, residues [12]. Another distinct feature of the NT-26 AioB Rieske structure is the absence of the disulphide bridge (C65–C80, *A. faecalis* numbering) shielding the cluster from solvent exposure (Figure 5). In NT-26 the aromatic ring of β F108 closes over the cluster packing against β G123 (Figure 5), and could thus play the role of the shield. In the *bc*_1_ complex Rieske protein the disulphide bridge has been suggested to be essential for the redox and catalytic properties of the 2Fe-2S cluster but its replacement by a F/G pair has not been tested [16], [18]. In NT-26 AioB S126 hydrogen bonds to the sulphur atom of the cluster as is the case in the *R. sphaeroides bc*_1_ complex whereas in *A. faecalis* AioB T83 is in the equivalent position (Figure 5). In the *R. sphaeroides bc*_1_ Rieske, substituting the S for the T decreased the redox potential of the cluster [15]. We reasoned these changes could account for the difference in the E_m_ value of the AioB Rieske clusters. The AioB proteins of NT-26 and *A. faecalis* have a F residue in common (F128 in NT-26 numbering) which is replaced with a Y in the *R. sphaeroides bc*_1_ Rieske. The substitution of the Y for a F in the *bc*_1_ Rieske cluster resulted in a decreased redox potential of the cluster and this could account for the difference in redox potentials of the NT-26 AioB and *bc*_1_ complex Rieske clusters. Site-directed mutagenesis experiments were performed to determine whether the structural variation in the Rieske clusters accounts for the observed differences in redox potentials previously reported [12].

### Effect of Mutations on the AioB Rieske Cluster on the Redox Potential of the Cluster

To try and understand the specific role of certain amino acids and the disulphide bridge in the AioB subunit three mutants were created: 1) S126 was mutated to a T to resemble the low redox potential (i.e. +130/160 mV) cluster of the *A. faecalis* enzyme, 2) F128 was mutated to a Y to resemble the *bc*_1_ complex Rieske with a high redox potential (i.e. +300 mV) and 3) F108 and G123 residues were mutated to C to introduce a disulphide bridge into the NT-26 Rieske cluster resembling the Rieske clusters of the *A. faecalis* AioB and the *bc*_1_ complex. All purified mutant enzymes were found to be heterotetramers. The theoretical mass of the β F108C/G123C mutant was calculated to be 17865.76 Da from amino acid sequence. The formation of a disulphide bond would be accompanied by a loss of two protons (2 Da). The presence of the disulphide was tested by analysing the intact β subunit in a denaturing electrospray ionization mass spectrometry experiment which determined the mass to be 17863.551 Da, thereby confirming disulphide bond formation (Figure S3).

A summary of enzyme activities and redox potentials are shown in Table 2. When DCPIP was used as the artificial electron acceptor only the S126T mutant showed a large reduction in specific activity which compares well with the reduced activity of the S/T mutant of the *R. sphaeroides bc*_1_ complex Rieske cluster [15]. Early reports [16], [41] suggested that one of the roles of the Rieske disulphide bridge is cluster stability. No effect on cluster stability as determined by specific activity over time or a comparison of the temperature profiles of the wild-type and the β F108C/G123C mutant were detected (Figure S4). The temperature profiles also compare well with that of the heterologously expressed Aio from *A. faecalis* that contains the disulphide bridge [23], with all three enzymes displaying a maximum activity at 65°C.

**Table 2:** Summary of specific activities and redox potentials of the Rieske clusters of the NT-26 wild type and mutant enzymes.

Enzyme	Specific activity (µmol^−1^ min^−1^ mg^−1^)^a^	E_m_ b (mV)	ΔE_m_ (mV)	ΔE_m_ expected in *bc*_1_ complex Rieske [15], [16]
WT	1.7	225±10		
S126T	0.6	205±10	−20	−26/−28
F128Y	1.5	225±10	0	+45/+70
F108C/G123C	1.5	190±10	−35	+54/+139^c^

a Average activity of at least two assays from two independent enzyme purifications.

b Data presented from one representative experiment. The results were the same in a separate experiment with independent enzyme preparations.

c The equivalent of this specific mutation has not been tested in the *bc*_1_ complex Rieske but all mutations removing the disulphide bridge result in a redox potential decrease [16], [18].

The redox potentials of the Rieske 2Fe-2S clusters have been evaluated on the EPR *g_y_* = 1.88 signal (Figure 5). EPR characterization of the wild type and mutant enzymes revealed a decrease in the redox potential of the Rieske cluster of the β S126T and β F108C/G123C mutants when compared to the wild type AioB and the β F128Y mutant (Table 2; redox titrations are shown in Figure S5). The β S126T mutant showed a similar E_m_ value decrease to the corresponding S/T mutation in the *bc*_1_ complex Rieske protein (Table 2). The E_m_ values of the β F108C/G123C and the β F128Y however are not comparable to those obtained for the equivalent mutants of the *bc*_1_ complex Rieske protein. The introduction of a disulphide bridge in the NT-26 AioB had a decreased E_m_ value rather than an increased one as would be expected for the *bc*_1_ complex Rieske. Previously, the presence or absence of the disulphide bridge in AioB has had no correlation to the E_m_ value [12]. The removal of this bridge in an AioB naturally harbouring it had no effect on the E_m_ value which suggested the absence of any role for this bridge in the redox properties of this cluster [42]. The result obtained with the NT-26 AioB F108C/G123C mutant suggests a specific role for the F108/G123 pair of residues in this protein and in other homologues also containing this F/G change (i.e. those from other arsenite-oxidising *Alphaproteobacteria*). The disulphide bridge is also absent in the putative arsenite oxidases from the hyperthermopilic *Archaea* (e.g. *Aeropyrum pernix* and *Pyrobaculum calidifontis*) which instead contain the residues glycine and leucine. The β F128Y mutation which we predicted would increase the redox potential of the 2Fe-2S cluster in the NT-26 AioB showed the same E_m_ as the wild type.

## Discussion

*Rhizobium* sp. NT-26, unlike *A. faecalis*, can oxidize arsenite autotrophically or heterotrophically obtaining energy from its oxidation [1]. In NT-26 the Aio is involved in this respiratory process where arsenite oxidation is coupled to the reduction of oxygen to water in an electron transport chain that involves a soluble *c*-type cytochrome [10].

Here we describe the expression of the Aio from NT-26 in the host *E. coli*. The expression of the enzyme compares well to the expression of other *bis*-MGD enzymes in *E. coli,* for example the *R. sphaeroides* DMSO or biotin sulphoxide (BSO) reductases [43], [44]. We have purified the Aio with a yield of 1.1 mg L^−1^ of *E. coli* culture with a *bis*-MGD saturation of approximately 83%. The yields of the DMSO and BSO reductases were 0.5 mg L^−1^ and 1.15 mg L^−1^, respectively with 90% and 88% *bis*-MGD saturation, respectively. In these studies, JM109 or BL21 cells were used as expression hosts and expression was performed under anaerobic conditions in a minimal medium. In contrast to other groups, we have used DH5α as the expression host, which when grown aerobically in a rich medium gave the highest enzyme yield. The K*_m_* of the recombinant enzyme was similar to that of the native enzyme but the V*_max_* was about 1.4-fold lower. Since EPR signals and redox potentials of the 3Fe-4S [42] and 2Fe-2S clusters show these centres to be correctly incorporated in the recombinant enzyme, the decreased V*_max_* may be in part explained by a proportion of the enzyme not containing the redox clusters as indicated by the metal analyses (Table S3).

The *A. faecalis* Aio was crystallized under two different conditions each with multiple heterodimers [6], the heterodimeric arrangement is identical to that of NT-26 (Figure S1). One crystal form (pH 6.4 1 g8k) when examined with PISA shows that the four heterodimers are arranged as two stable heterotetramers. The arrangement of the heterotetramer is very close to that of the NT-26 Aio and buries a similar amount of surface area. The other crystal form of *A. faecalis* Aio (pH 8.5, 1 g8j) has two heterodimers but analysis by PISA shows no stable heterotetramer. In fact, if one re-examines the crystal structure a similar heterotetrameric arrangement is seen as a result of crystal packing but the two dimers are separated and slightly rotated (in essence less tightly packed). Examining this arrangement in PISA suggests that this heterotetramer buries almost 3000 Å^2^ less surface area as a result of the separation. Consequently PISA analysis does not identify in this crystal the heterotetramer as stable. For the NT-26 Aio, biochemical data suggests that the heterotetramer is stable in solution and the functional unit. The data for the *A. faecalis* Aio are less clear cut with a heterodimer being regarded as the functional unit. However, the conservation of the tetrameric arrangement in three different crystals, with two crystal forms showing stable arrangements with extensive buried surface area, argues that the heterotetramer is most likely to be the functional unit for the Aio. In fact, we have recently demonstrated that the recombinant version of the *A. faecalis* Aio expressed in *E. coli* is a heterotetramer as determined by gel filtration chromatography (Heath & Santini, unpublished data). The multiple arrangement and apparent variability in heterotetramer strength (as judged by PISA) in *A. faecalis* Aio indicates that the heterodimer-heterodimer interface is flexible. The Mo metal sits in a five coordinate environment; four ligands come from the two dithiolene moieties, each of which comes from a pterin molecule. These four sulphur atoms sit in a plane with the Mo at the centre but slightly displaced out of the plane. The fifth site is occupied by an oxygen (presumably an oxo group) giving rise to a square pyramidal arrangement of the five ligands. The oxidation state of the Mo atom is not known although five coordinate Mo usually favours a +IV oxidation state, which has been identified in related enzymes. If correct then this would give an overall charge of −2. Reduction from the +VI state may have occurred during data collection (as proposed for the *A. faecalis* enzyme) or the enzyme may have a five coordinate +VI oxidation state. There is no evidence in the NT-26 Aio for a sixth ligand to the Mo centre. In the *A. faecalis* Aio Ellis *et al*. [6] proposed the presence of a hydroxide in the +VI state, although this gave an unusual geometry which would require some re-arrangement of the protein. The second coordination sphere around the Mo is identical in both the *A. faecalis* and NT-26 AioA subunits, comprising H199, N200, R201, E207, K385, R447 and H451 (NT-26 numbering). These residues are predicted to control the recognition and orientation of the incoming arsenite.

Several residues surrounding the 3Fe-4S centre are different between the *A. faecalis* and NT-26 enzymes. One of them, S98 (in *A. faecalis*) is not conserved between alpha- and betaproteobacterial AioA. In NT-26, and all the alphaproteobacterial AioA, this residue is replaced by a G [42]. The *A. faecalis* structure features a hydrogen bond between the Rieske and the 3Fe-4S cluster mediated by this S98 together with the adjacent conserved S99. There is no such hydrogen bond between G and S in the NT-26 Aio structure. The presence of this hydrogen bond could have an effect on redox interactions between both Fe-S clusters as proposed previously [42].

**Figure 6:**
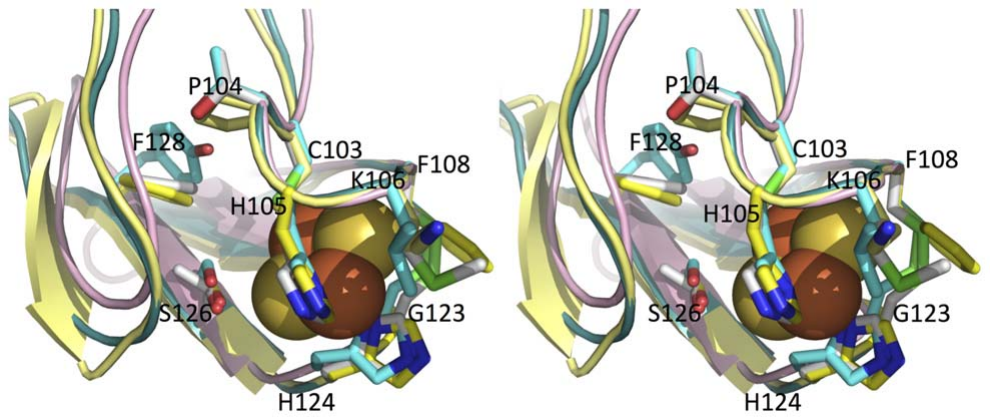
Close up view of the Aio Rieske 2Fe-2S cluster. A wall eye stereo superposition of NT-26 (yellow cartoon, with sticks having yellow coloured carbon atoms), *A. faecalis* (teal cartoon, with sticks having white coloured carbon atoms) and *R. sphaeroides* (2qjk) *bc*_1_ complex (salmon cartoon, with sticks having cyan coloured carbon atoms). Nitrogen atoms are coloured blue, oxygen atoms coloured red and sulphur atoms green when shown in stick in all structures. The Rieske cluster from the NT-26 structure is shown with iron atoms as brown spheres and sulphur atoms as dull yellow spheres. The residues in the NT-26 are labelled and the corresponding atoms in the other structures are discussed in the text. The superposition was generated by using all backbone atoms from residue 104 to residue 110 in the NT-26 Aio structure as the template. This provides a more meaningful view of changes at the Rieske cluster than a simple all atom superposition.

The redox potentials of the Rieske 2Fe-2S clusters of the native NT-26 and *A. faecalis* AioB subunits differ significantly (+225 mV *vs. +*130/160 mV, respectively). As shown in Figure S1, variation in overall fold does not account for this difference but several residues are distinct in the two enzymes (Figure 6). In place of the β S126 in NT-26, the *A. faecalis* AioB contains a T. The E_m_ value decrease of 20 mV, observed with the NT-26 β S126T mutant is similar to the measured decrease in the *bc*_1_ complex Rieske mutants of *R. sphaeroides* (i.e. S154T) [15], [17] or *Saccharomyces cerevisiae* (i.e. S163T) [45]. The superimposed structures of the Rieske subunits show that the S or T are overlapping in the AioB of *A. faecalis* and NT-26 with that of the *R. sphaeroides bc*_1_ complex (Figure 6). The remaining difference between the E_m_ value of the NT-26 and *A. faecalis* AioB could be due to specific replacement of the disulphide bridge by the F/G pair. In fact, this difference in E_m_ value is in good agreement with the observed decrease of E_m_ value when mutating F/G to C/C in the NT-26 AioB. The bulky F residue, could shield the cluster more from the water better than the small C which is supported by the observation that the replacement of the C by a small A doesn’t affect the redox potential of the *Ralstonia* sp. S22 AioB Rieske cluster [42]. The absence of any effect of the F128Y mutation on the NT-26 AioB was surprising. This mutation had been extensively studied in the Rieske of the *bc*_1_ complex [14]–[17], [45] and has been proposed to account for the difference in E_m_ value between AioB in general and the high redox potential of the *bc*_1_ complex Rieske clusters [12]. A detailed examination of the superimposed structures highlights a distinct torsion of the beta sheet surrounding the Rieske cluster in AioB compared to the *bc*_1_ complex Rieske. This torsion results in an increased distance of 1 Å between the F128 residue and the sulphur atom of the cluster-ligating C residue (C103, NT-26 numbering) compared to the *R. sphaeroides bc*_1_ complex Rieske Y156F mutant. This explains why the introduction of a Y in the NT-26 AioB had no effect on the redox potential of the cluster as the distance is too great from C103 for the formation of a hydrogen bond.

## Conclusions

In this study we have determined the optimal conditions for the heterologous expression of the first Aio from an autotrophic arsenite-oxidising. Structural studies have demonstrated a high degree of similarity to the Aio from *A. faecalis* which is thought to oxidise arsenite for detoxification [8]. There are also some striking differences in the Aio structures particularly in the region surrounding the AioB 2Fe-2S cluster. By using a combination of site-directed mutagenesis and EPR we have explained why the differences observed in the redox potentials of the Rieske subunit of the Aio and *bc*_1_ complex exist.

## Supporting Information

Figure S1 The heterodimeric structure of NT-26 Aio superimposed on that of *A. faecalis.* The folding of the NT-26 Aio is essentially identical to that of *A. faecalis* [α chain (marine blue) and β chain (pale cyan)]. The *A. faecalis* coordinates are taken from 1G8K.
(TIF)

Figure S2 The four domains of the large catalytic arsenite oxidase subunit, AioA. Domain 1 is coloured in dark blue, domain 2 in red, with the additional small domain in salmon, domain 3 in cyan and domain 4 in green. The structure has a pseudo tetrahedral arrangement. Domains 2 and 3 can be superimposed as they share a similar fold.
(TIF)

Figure S3 [Main figure] denatured spectrum of β F108C/G123C mutant. [Inset] MaxEnt deconvolution showing the masses found. The calculated mass of AioB with a disulphide bond is 17863.76 Da, which is indicated by the peak at 17863.551 Da. The 18039.301 Da peak is the AioB subunit bound to the 2Fe-2S cluster, and the 17928.150 Da peak is co-purified protein.
(TIF)

Figure S4 Temperature-activity profiles of the NT-26 wild-type and β F108C/G123C mutant arsenite oxidases. Percentage of maximum activity is plotted as a function of temperature of WT (•) and β F108C/G123C (○) Aio. Data points and error bars represent mean and standard deviation of at least three assays, respectively.
(TIF)

Figure S5 Potentiometric titrations of the Rieske iron-sulphur cluster of the wild-type and mutant NT-26 arsenite oxidases. Potentiometric titrations were performed at pH 8 following the *g* = 1.88 signal. The data for the titration of the wild-type enzyme are represented with solid squares. The related fit is in a straight line. The data for the titration of the F128Y mutant are represented with open triangles. The related fit is shown as a dotted line. The data for the titration of the S126T enzyme are represented with solid circles. The related fit is shown as a straight line. The data for the titration of the F108C/G123C mutant are represented with solid inverted triangles. The related fit is shown as a straight line.
(TIF)

Table S1 Primers used for site-directed mutagenesis of *aioB.*
(TIF)

Table S2 Crystallographic data.
(TIF)

Table S3 Determination of the molybdenum, iron and nucleotide content of the recombinant NT-26 arsenite oxidase. ^a^Molybdenum (µM molybdenum/µM Aio) and iron (µM iron in relation to 1×[3Fe-4S] and 1×[2Fe2S]/µM Aio) content were determined by ICP-OES (PerkinElmer Optima 2100DV, Fremont, CA, USA). Results are related to one catalytic subunit (i.e. αβ AioBA heterodimer). ^b^Nucleotide content (µM CMP or AMP or GMP/µM Aio) was analysed after release of nucleotide from the molybdenum cofactor by heat treatment under acidic conditions. AMP, CMP and GMP were quantified relative to AMP, CMP and GMP standard solutions. ^c^No nucleotide detected.
(TIF)
