# Supplementary figures and images for: The Respiratory Arsenite Oxidase: Structure and the Role of Residues Surrounding the Rieske Cluster
Source: PLoS One. 2013 Aug 30;8(8):e72535. doi: 10.1371/journal.pone.0072535 (PMC3758308; doi:10.1371/journal.pone.0072535)

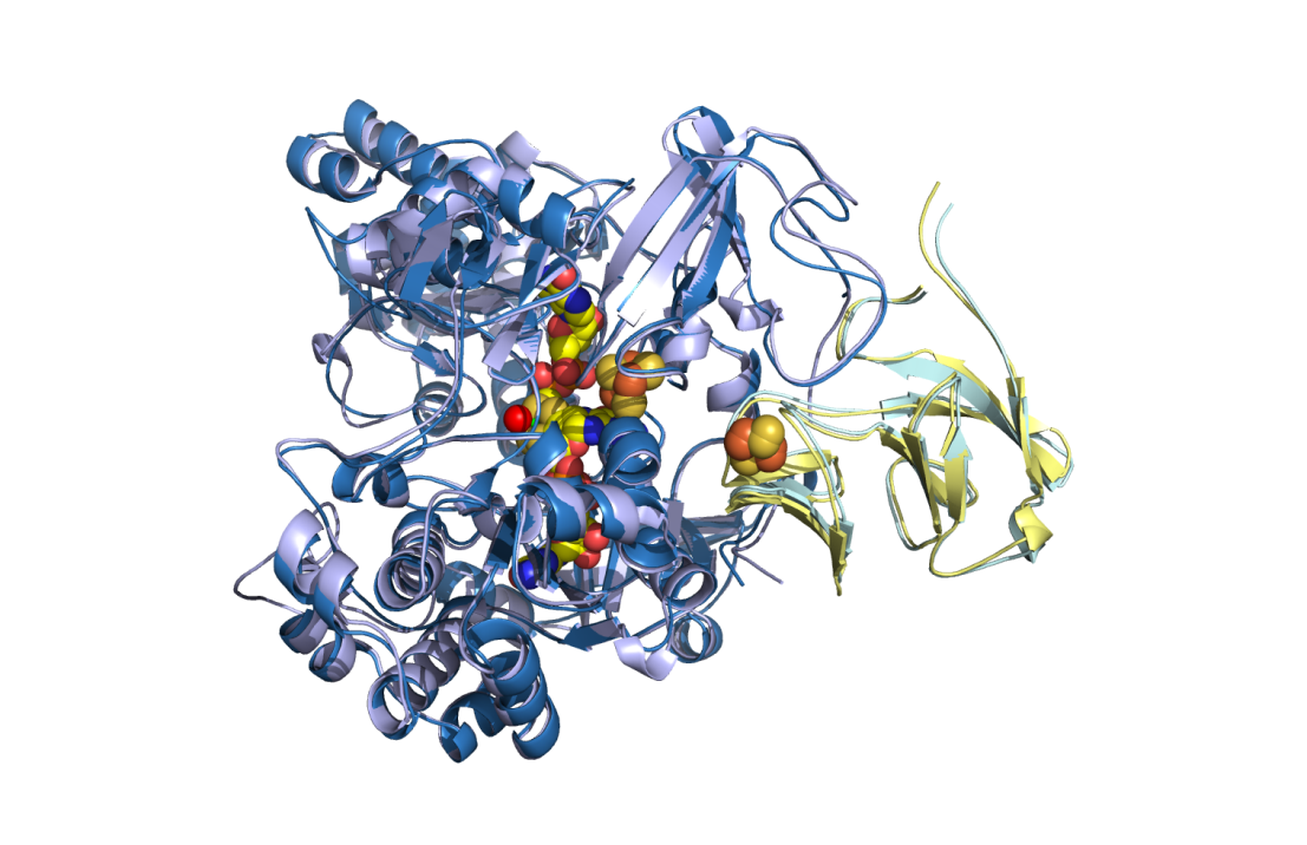

Supplement: Figure S1 — The heterodimeric structure of NT-26 Aio superimposed on that of A. faecalis. The folding of the NT-26 Aio is essentially identical to that of A. faecalis [α chain (marine blue) and β chain (pale cyan)]. The A. faecalis coordinates are taken from 1G8K. (TIF) [file pone.0072535.s001.tif]

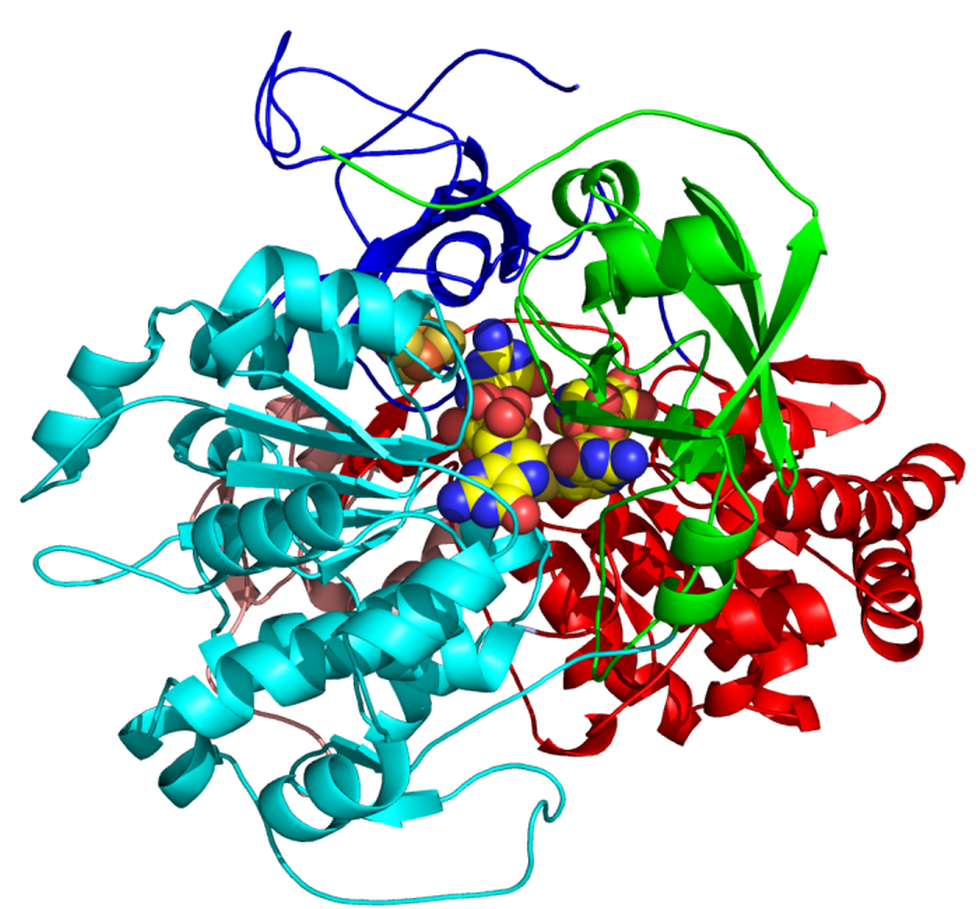

Supplement: Figure S2 — The four domains of the large catalytic arsenite oxidase subunit, AioA. Domain 1 is coloured in dark blue, domain 2 in red, with the additional small domain in salmon, domain 3 in cyan and domain 4 in green. The structure has a pseudo tetrahedral arrangement. Domains 2 and 3 can be superimposed as they share a similar fold. (TIF) [file pone.0072535.s002.tif]

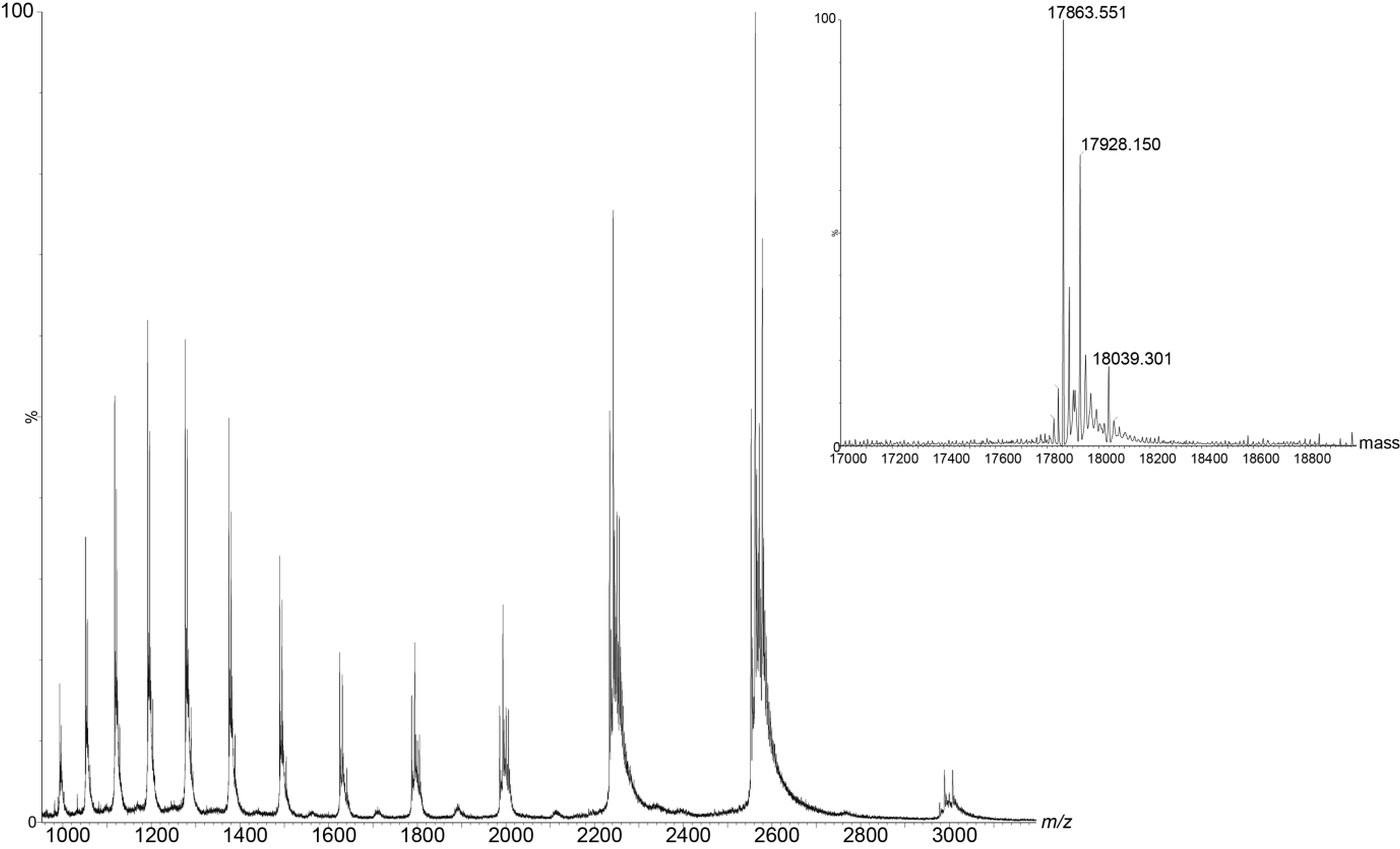

Supplement: Figure S3 — [Main figure] denatured spectrum of β F108C/G123C mutant. [Inset] MaxEnt deconvolution showing the masses found. The calculated mass of AioB with a disulphide bond is 17863.76 Da, which is indicated by the peak at 17863.551 Da. The 18039.301 Da peak is the AioB subunit bound to the 2Fe-2S cluster, and the 17928.150 Da peak is co-purified protein. (TIF) [file pone.0072535.s003.tif]

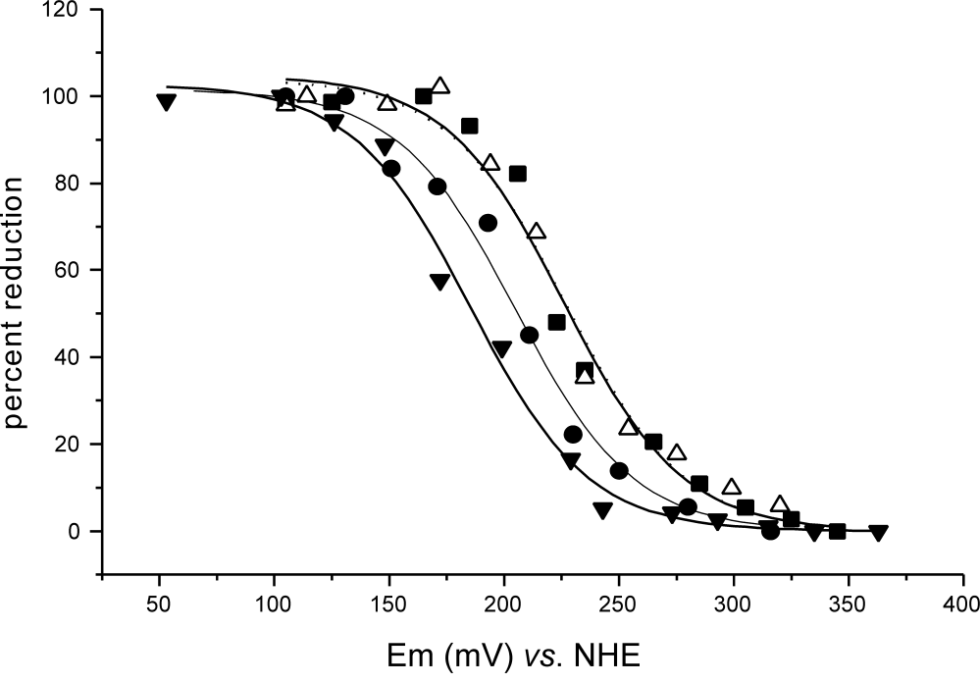

Supplement: Figure S5 — Potentiometric titrations of the Rieske iron-sulphur cluster of the wild-type and mutant NT-26 arsenite oxidases. Potentiometric titrations were performed at pH 8 following the g = 1.88 signal. The data for the titration of the wild-type enzyme are represented with solid squares. The related fit is in a straight line. The data for the titration of the F128Y mutant are represented with open triangles. The related fit is shown as a dotted line. The data for the titration of the S126T enzyme are represented with solid circles. The related fit is shown as a straight line. The data for the titration of the F108C/G123C mutant are represented with solid inverted triangles. The related fit is shown as a straight line. (TIF) [file pone.0072535.s005.tif]

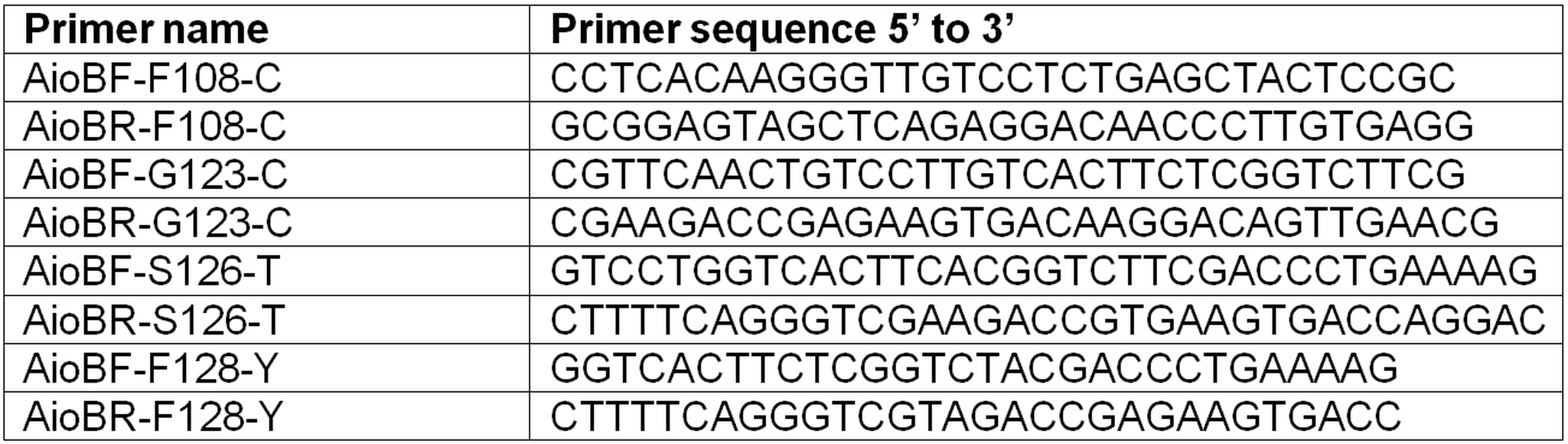

Supplement: Table S1 — Primers used for site-directed mutagenesis of aioB. (TIF) [file pone.0072535.s006.tif]

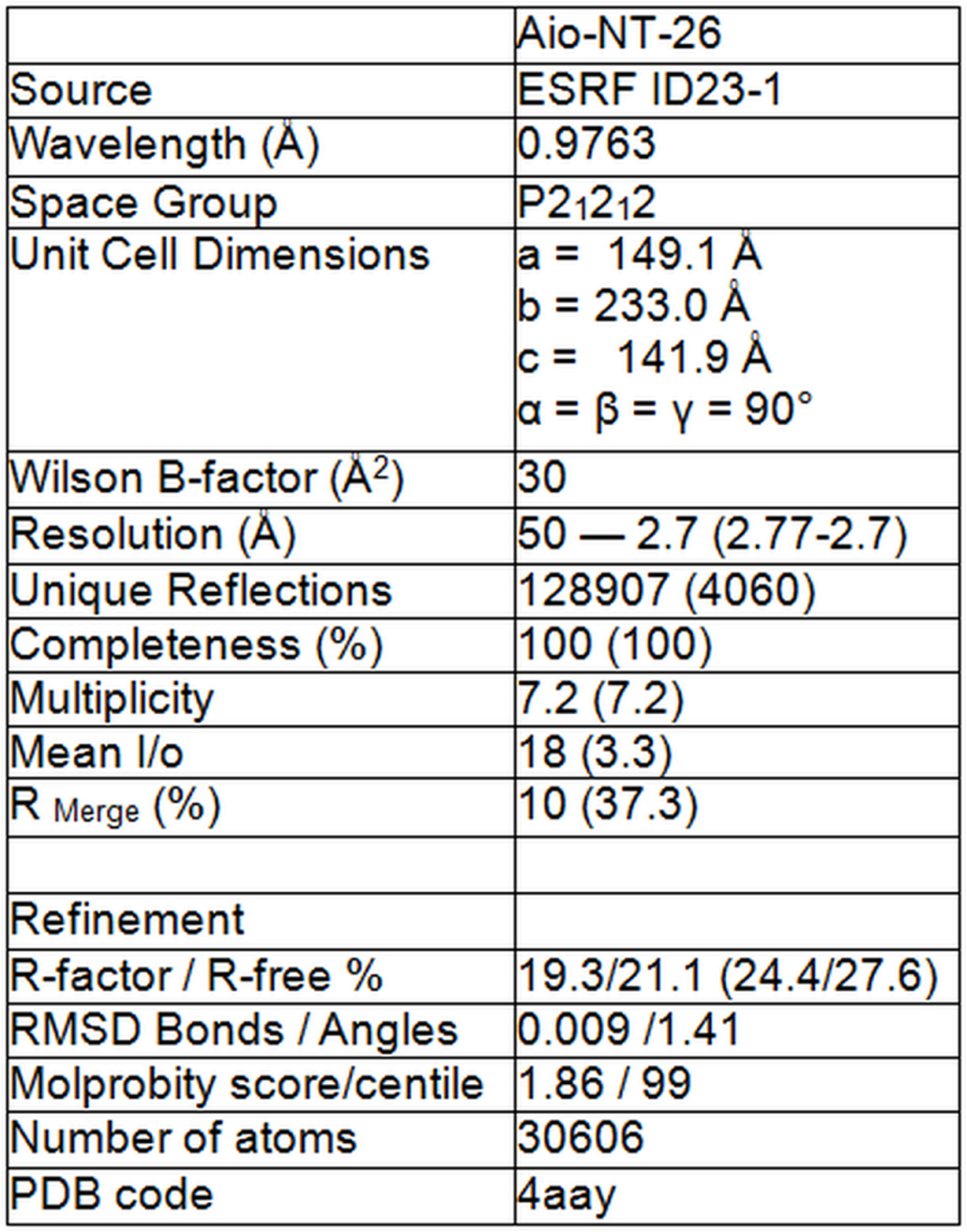

Supplement: Table S2 — Crystallographic data. (TIF) [file pone.0072535.s007.tif]

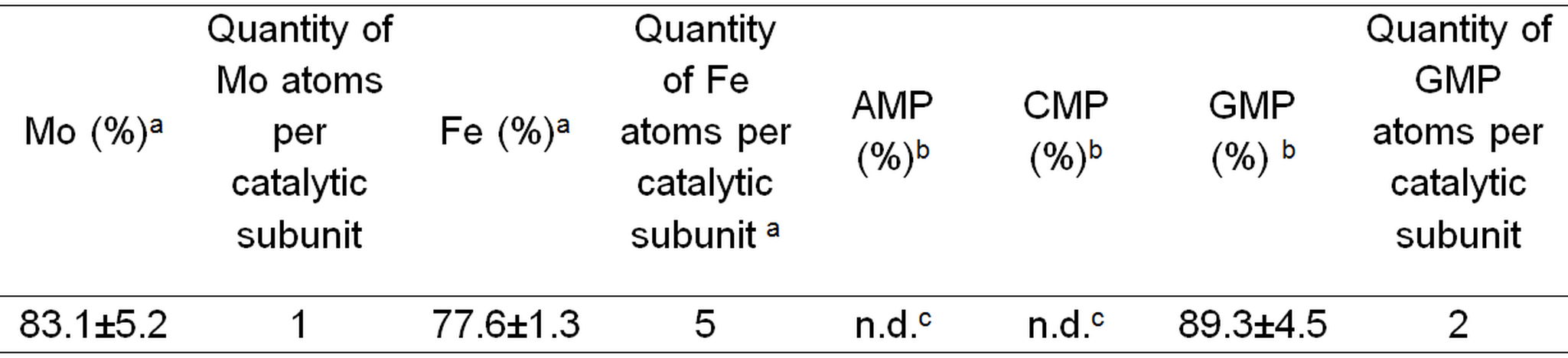

Supplement: Table S3 — Determination of the molybdenum, iron and nucleotide content of the recombinant NT-26 arsenite oxidase. aMolybdenum (µM molybdenum/µM Aio) and iron (µM iron in relation to 1×[3Fe-4S] and 1×[2Fe2S]/µM Aio) content were determined by ICP-OES (PerkinElmer Optima 2100DV, Fremont, CA, USA). Results are related to one catalytic subunit (i.e. αβ AioBA heterodimer). bNucleotide content (µM CMP or AMP or GMP/µM Aio) was analysed after release of nucleotide from the molybdenum cofactor by heat treatment under acidic conditions. AMP, CMP and GMP were quantified relative to AMP, CMP and GMP standard solutions. cNo nucleotide detected. (TIF) [file pone.0072535.s008.tif]
